# Prognostic stratification through smoking status and cumulative smoking dose in advanced non-small cell lung cancer immunotherapy: a dose-dependent real-world analysis

**DOI:** 10.3389/fonc.2025.1590825

**Published:** 2025-08-06

**Authors:** Lan Yang, Haoyu Wang, Panwen Tian, Weimin Li

**Affiliations:** ^1^ Department of Pulmonary and Critical Care Medicine State Key Laboratory of Respiratory Health and Multimorbidity West China Hospital of Sichuan University, Precision Medicine Key Laboratory of Sichuan Province, Chengdu, Sichuan, China; ^2^ Institute of Respiratory Health, Frontiers Science Center for Disease-related Molecular Network, West China Hospital, Sichuan University, Chengdu, Sichuan, China; ^3^ Lung Cancer Center/Lung Cancer Institute, West China Hospital, Sichuan University, Chengdu, China

**Keywords:** non-small cell lung cancer, immune checkpoint inhibitors, smoking hygiene, cumulative smoking dose, real-world evidence

## Abstract

**Background:**

The association between smoking status, cumulative smoking dose, and immunotherapy efficacy in non-small cell lung cancer (NSCLC) remains controversial. We sought to integrate the lifetime pack-years with smoking cessation status to identify optimal immunotherapy beneficiaries.

**Methods:**

A total of 1,192 immunotherapy-treated NSCLC patients treated between November 2015 and April 2024 were enrolled. Data on demographics, clinical characteristics, pathologic characteristics, treatments, and clinical outcomes were collected. The objective response rate (ORR), disease control rate (DCR), and progression-free survival (PFS) were compared across different smoking statuses (never, current, and former smokers) and cumulative smoking doses (never smokers, non-heavy smokers: <20 pack-years, and heavy smokers: ≥20 pack-years). Multivariate logistic regression and Cox proportional hazards models were used to analyze ORR and PFS, respectively.

**Results:**

Among the 1,192 patients, 377 were never smokers, 499 were current smokers, and 316 were former smokers. In terms of smoking status, former smokers exhibited the longest median PFS (17.0 months, *P* < 0.001), with the highest ORR (46.8%, *P* < 0.001) and DCR (86.7%, *P* = 0.008). Regarding cumulative smoking dose, the heavy smoker group demonstrated the longest median PFS (15.9 months, *P* = 0.001), with the highest ORR (46.6%, *P* < 0.001) and DCR (85.2%, *P* = 0.012). Notably, further multivariate analysis identified former heavy smokers as independent favorable predictors of ORR (OR = 1.93, 95% CI = 1.25–2.99, *P* = 0.003) and PFS (HR = 0.75, 95% CI = 0.57–0.99, *P* = 0.04) in advanced NSCLC patients receiving immunotherapy.

**Conclusions:**

This real-world cohort analysis establishes a clinical stratification combining smoking cessation status with cumulative smoking dose, identifying former heavy smokers as optimal immunotherapy beneficiaries. These findings advocate integrated smoking history documentation and emphasize clinical prioritization of cessation interventions to enhance treatment efficacy in NSCLC.

## 1 Introduction

Lung cancer is the leading cause of cancer-related mortality worldwide (1), with non-small cell lung cancer (NSCLC) representing approximately 85% of lung cancer cases (2). Due to the lack of prominent symptoms in early-stage NSCLC, most patients are diagnosed at advanced stages when symptoms become apparent, with a 5-year survival rate of only 27% (1). In recent years, the introduction of immune checkpoint inhibitors (ICIs), particularly targeting cytotoxic T-lymphocyte-associated protein 4 (CTLA-4), programmed cell death protein 1 (PD-1), and programmed cell death-ligand 1 (PD-L1), has led to significant breakthroughs in the treatment of advanced NSCLC (3). The immunotherapy has notably improved the prognosis of NSCLC patients with advanced disease and has become one of the standard treatment strategies in international lung cancer guidelines.

Despite these advancements, the clinical response to immunotherapy varies among individuals, with fewer than 20% of unselected NSCLC patients benefiting from anti-PD-1/PD-L1 therapies (4, 5). Therefore, identifying biomarkers that can assist in selecting patients who would benefit the most from immunotherapy is of critical importance. The PD-L1 expression level is one of the earliest indicators used to predict the effect of immunotherapy and select the target population. Compared to those with low or negative PD-L1 expression, patients with high PD-L1 expression in advanced NSCLC are more likely to benefit from immunotherapy, exhibiting higher objective response rates (ORRs) (6–8). However, its clinical utility remains limited, and not all patients with a high tumor proportion score (TPS) for PD-L1 expression experience clinical benefit (9). Other biomarkers related to the prediction of immunotherapy efficacy include tumor mutation burden (TMB), tumor-infiltrating lymphocytes (TILs), microsatellite instability (MSI), tumor genomic characteristics, and so on (9–11).

Smoking is a well-established risk factor for the development of NSCLC and is also thought to increase the overall risk of patients dying from cancer (12). With the development of tumor immunotherapy, several studies have suggested that current and former smokers may experience improved prognosis with immunotherapy (13), while a recent study argues that never smokers may exhibit increased progression-free survival (PFS) and overall survival (OS) (14). Nevertheless, the meta-analysis involving 17 phase III trials with 10,283 patients suggested that smoking status does not significantly affect the efficacy of immunotherapy (15).

In this study, we aimed to investigate the effect of smoking status and cumulative smoking dose on clinical outcomes in patients with advanced NSCLC undergoing immunotherapy.

## 2 Materials and methods

### 2.1 Study design and participants

This retrospective study included adult patients (≥18 years) who were histologically diagnosed with primary advanced NSCLC, including pathological stages III and IV, at West China Hospital of Sichuan University between November 2015 and April 2024. All included patients were confirmed via pathological diagnosis and received either ICI monotherapy or a combination of ICI and chemotherapy. Patients were excluded if they had 1) a history of other malignancies or 2) incomplete data on smoking history. The study was approved by the Medical Ethics Committee of West China Hospital of Sichuan University (approval number: 2024-1410), and the requirement for informed consent was waived because the data were deidentified.

### 2.2 Data collection

We collected the following data from the patients: gender, age at diagnosis, smoking history, body mass index (BMI), Eastern Cooperative Oncology Group Performance Status (ECOG PS), histological type of the tumor, the tumor–node–metastasis (TNM) stage, programmed cell death ligand 1 (PD-L1) expression levels, the combination therapy (including chemotherapy and radiotherapy), and lines of treatment. The categories of BMI were defined as follows: underweight, BMI less than 18.5; normal, BMI 18.5 to less than 24.9; and overweight or obese, BMI 25 or higher (16). The TNM stage was classified according to the 8th edition International Association for the Study of Lung Cancer (IASLC) staging system for lung cancer (17). PD-L1 expression was assessed using 22C3 pharmDx immunohistochemistry antibody and classified into three categories: tumor proportion score (TPS) <1% (negative), TPS 1%–49% (low expression), and TPS ≥50% (high expression) (18).

Never smokers were defined as individuals who had smoked fewer than 100 cigarettes in their lifetime (19). Current smokers were individuals who smoked regularly or occasionally, and former smokers were those who had quit smoking. Current and former smokers were categorized as ever smokers. The number of pack-years of cigarette smoking was calculated by multiplying the average number of cigarettes smoked per day by the total number of years the individual has smoked and then dividing this product by 20 (average number of cigarettes in a pack). Individuals were classified as non-heavy smokers if they had accumulated fewer than 20 pack-years of smoking, while those with 20 or more pack-years were categorized as heavy smokers (20).

### 2.3 Outcomes

The primary endpoint was PFS, defined as the time from the initiation of immunotherapy to disease progression or death from any cause (21). Secondary endpoints included ORR, defined as the proportion of patients achieving a complete response (CR) or partial response (PR) to treatment, and disease control rate (DCR), defined as the proportion of patients with CR, PR, or stable disease (SD). The efficacy of immunotherapy was evaluated by experienced clinicians according to the Response Evaluation Criteria in Solid Tumors (RECIST) version 1.1 (22). The follow-up deadline was on 30 December 2024.

### 2.4 Statistical analysis

Categorical variables were summarized as frequencies and percentages, with group differences assessed using chi-square tests. The Fisher’s exact test was used when expected frequencies were low. PFS was estimated using the Kaplan–Meier survival analysis, with the subgroup differences compared using log-rank tests. Logistic regression and Cox proportional hazards regression were employed to identify potential factors influencing ORR and PFS in advanced NSCLC, respectively. Variables with a *P*-value <0.05 in the univariate analysis were included in the multivariate model. All statistical analyses were performed using R version 4.3.3 (R Foundation, Vienna, Austria), with statistical significance defined as a two-tailed *P*-value <0.05.

## 3 Results

### 3.1 Baseline characteristics

A total of 1,192 patients with advanced NSCLC who received immunotherapy between November 2015 and April 2024 were included in this study. Of these, 377 were never smokers, 499 were current smokers (median pack-years, 40), and 316 were former smokers (median pack-years, 30). The baseline characteristics of the patients are summarized in **Table 1**. The vast majority of the patients were men (974, 81.7%), and approximately 54.9% were aged 60 years or older. Of the total number of patients, 9.0% had a BMI less than 18.5 kg/m^2^, 23.0% had a BMI greater than 25 kg/m^2^, and 92.5% had an ECOG PS of 0 or 1. Stage IV disease was diagnosed in 68.5% of patients. In terms of histological subtypes, 48.4% had adenocarcinoma, 43.0% had squamous cell carcinoma (SCC), and 8.6% had other subtypes. Regarding PD-L1 expression levels, 15.4% were negative, 23.8% had low expression, and 20.2% had high expression. Of the patients, 61.4% received first-line immunotherapy, 82.1% received chemotherapy, and 11.0% received radiotherapy. Significant differences in age, sex, TNM stage, histological subtypes, and treatment line were observed among the three groups.

**Table 1:** Clinicopathological characteristics among patients stratified by smoking status.

Characteristic	Overall	Never smoker	Current smoker	Former smoker	*P*-value
	*N* = 1,192	*N* = 377	*N* = 499	*N* = 316	
Gender, *n* (%)					<0.001
Female	218 (18.3)	213 (56.5)	1 (0.2)	4 (1.3)	
Male	974 (81.7)	164 (43.5)	498 (99.8)	312 (98.7)	
Age, *n* (%)					<0.001
<60	538 (45.1)	214 (56.8)	204 (40.9)	120 (38.0)	
≥60	654 (54.9)	163 (43.2)	295 (59.1)	196 (62.0)	
BMI, *n* (%)					0.14
<18.5	107 (9.0)	38 (10.1)	48 (9.6)	21 (6.6)	
18.5–24.9	811 (68.0)	242 (64.2)	350 (70.1)	219 (69.3)	
≥25	274 (23.0)	97 (25.7)	101 (20.2)	76 (24.1)	
ECOG PS, *n* (%)					0.10
0–1	1,103 (92.5)	340 (90.2)	469 (94.0)	294 (93.0)	
≥2	89 (7.5)	37 (9.8)	30 (6.0)	22 (7.0)	
Pack-years, median (IQR)	23 (0 – 40)	0	40 (25 – 50)	30 (20 – 40)	<0.001
Stage, *n* (%)					<0.001
III	376 (31.5)	83 (22.0)	182 (36.5)	111 (35.1)	
IV	816 (68.5)	294 (78.0)	317 (63.5)	205 (64.9)	
Histology, *n* (%)					<0.001
LUAD	578 (48.4)	232 (61.5)	215 (43.1)	131 (41.5)	
LUSC	512 (43.0)	108 (28.6)	242 (48.5)	162 (51.3)	
Others	102 (8.6)	37 (9.8)	42 (8.4)	23 (7.3)	
PD-L1, *n* (%)					0.83
<1%	184 (15.4)	57 (15.1)	76 (15.2)	51 (16.1)	
1%–49%	284 (23.8)	88 (23.3)	119 (23.8)	77 (24.4)	
≥50%	241 (20.2)	68 (18.0)	107 (21.4)	66 (20.9)	
Not assessed	483 (40.5)	164 (43.5)	197 (39.5)	122 (38/6)	
Treatment line, *n* (%)					<0.001
1st	732 (61.4)	191 (50.7)	353 (70.7)	188 (59.5)	
More than 2nd	460 (38.6)	186 (49.3)	146 (29.3)	128 (40.5)	
Chemotherapy, *n* (%)	979 (82.1)	315 (83.6)	410 (82.2)	254 (80.4)	0.55
Radiotherapy, *n* (%)	131 (11.0)	47 (12.5)	58 (11.6)	26 (8.2)	0.17

BMI, body mass index; ECOG PS, Eastern Cooperative Oncology Group Performance Status; IQR, interquartile range; LUAD, lung adenocarcinoma; LUSC, lung squamous cell carcinoma; PL-L1, programmed cell death-ligand 1.

### 3.2 Immunotherapy efficacy stratified by smoking status

We first examined the relationship between ORR and smoking status among patients. The overall ORRs were 30.0% for never smokers, 46.1% for current smokers, and 46.8% for former smokers, with a statistically significant difference (*P* < 0.001). DCR was also the highest in former smokers at 86.7%, compared to 78.0% in never smokers and 83.6% in current smokers (*P* = 0.008) (**Table 2**).

**Table 2:** Efficacy outcomes stratified by smoking status.

Clinical response	Overall	Never smokers	Current smokers	Former smokers	*P*-value
	*N* = 1,192	*N* = 377	*N* = 499	*N* = 316	
CR	3 (0.3)	2 (0.5)	0 (0)	1 (0.3)	0.27
PR	488 (40.9)	111 (29.4)	230 (46.1)	147 (46.5)	<0.001
SD	494 (41.4)	181 (48.0)	187 (37.5)	126 (39.9)	0.006
PD	207 (17.4)	83 (22.0)	82 (16.4)	42 (13.3)	0.008
ORR, *n* (%)					<0.001
Non-response	701 (58.8)	264 (70.0)	269 (53.9)	168 (53.2)	
Response	491 (41.2)	113 (30.0)	230 (46.1)	148 (46.8)	
DCR, *n* (%)					0.008
Non-response	207 (17.4)	83 (22.0)	82 (16.4)	42 (13.3)	
Response	985 (82.6)	294 (78.0)	417 (83.6)	274 (86.7)	
PFS					<0.001
Median (95% CI), months	13.6 (12.2, 15.4)	11.2 (9.8, 12.8)	14.9 (12.7, 16.9)	17.0 (12.8, 23.0)	
% at 12 months (95% CI)	53.8 (50.6, 57.1)	46.1 (40.8, 52.1)	56.7 (51.9, 61.9)	58.6 (52.7, 65.2)	
% at 36 months (95% Cl)	27.6 (24.2, 31.3)	22.6 (17.2, 28.8)	26.0 (21.0, 32.3)	36.6 (30.2, 44.4)	
% at 60 months (95% CI)	18.8 (14.4, 24.5)	11.4 (5.5, 23.6)	16.7 (10.5, 26.4)	29.5 (21.2, 41.1)	

CR, complete response; PR, partial response; SD, stable disease; PD, progressive disease; ORR, objective response rate; DCR, disease control rate; PFS, progression-free survival; CI, confidence interval.

**Figure 1:**
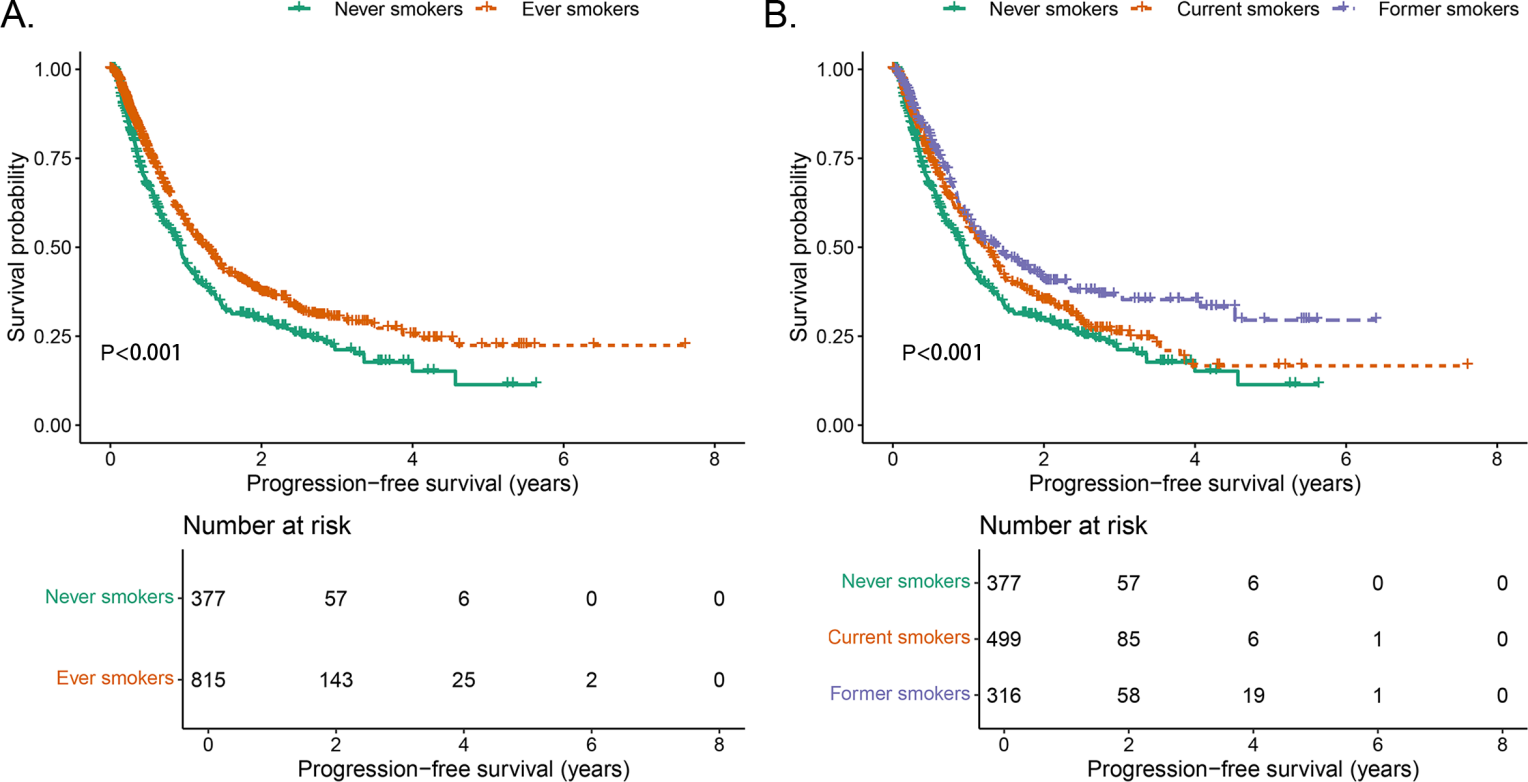
Comparison of PFS stratified by smoking status. **(A)** Comparison of PFS between never smokers and ever smokers. **(B)** Comparison of PFS between never smokers, current smokers, and former smokers.

In never smokers, the median PFS was 11.2 months (95% CI: 9.8–12.8), with 1-, 3-, and 5-year PFS rates of 46.1% (95% CI: 40.8–52.1), 22.6% (95% CI: 17.2–28.8), and 11.4% (95% CI: 5.5–23.6), respectively. Compared with never smokers, ever smokers (defined as individuals who currently or formerly smoked) exhibited significantly longer PFS (*P* < 0.001, **Figure 1A**). Further stratifying the ever-smoker group, current smokers had a median PFS of 14.9 months (95% CI: 12.7–16.9), with 1-, 3-, and 5-year PFS rates of 56.7% (95% CI: 51.9–61.9), 26.0% (95% CI: 21.0–32.3), and 16.7% (95% CI: 10.5–26.4), respectively. Former smokers had the longest median PFS at 17.0 months (95% CI: 12.8–23.0, *P* < 0.001), with 1-, 3-, and 5-year PFS rates of 58.6% (95% CI: 52.7–65.2), 36.6% (95% CI: 30.2–44.4), and 29.5% (95% CI: 21.2–41.1), respectively (**Table 2**, **Figure 1B**).

### 3.3 Immunotherapy efficacy stratified by both smoking status and cumulative smoking dose

**Figure 2:**
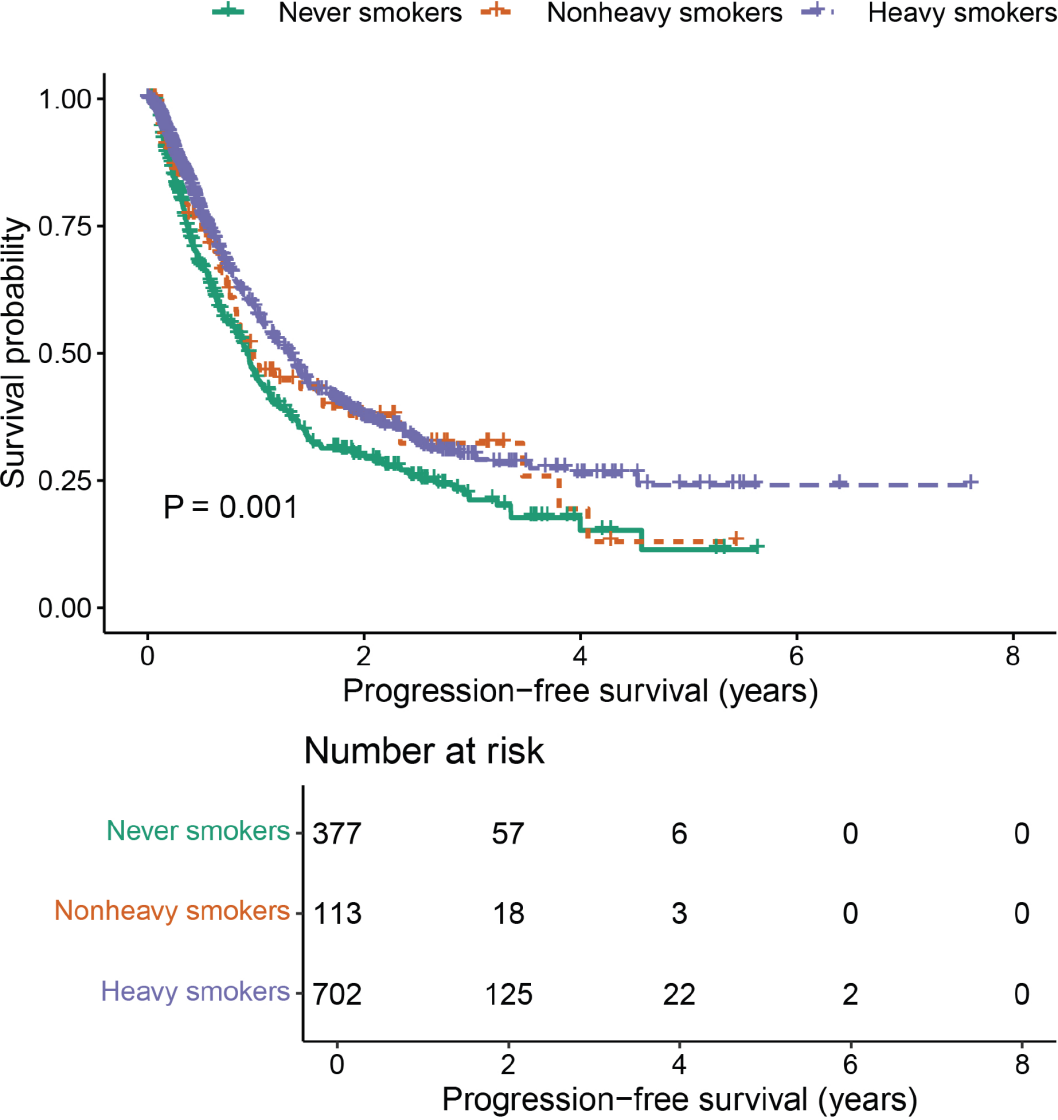
Comparisons of PFS stratified by cumulative smoking dose.

A positive correlation was observed between efficacy outcomes and cumulative smoking dose. The ORR in never smokers was 30.0%. As the cumulative smoking dose increased, ORR showed a significant improvement (*P* < 0.001, **Table 2**), rising to 45.1% in non-heavy smokers and 46.6% in heavy smokers. A similar trend was seen in DCR, with values of 78.0%, 82.3%, and 85.2% in never smokers, non-heavy smokers, and heavy smokers, respectively (*P* = 0.012, **Table 2**). Additionally, cumulative smoking dose exhibited a dose-dependent positive correlation with median PFS, which was statistically significant (*P* = 0.001, **Table 3**, **Figure 2**).

**Table 3:** Efficacy outcomes stratified by cumulative smoking dose.

Characteristic	Overall	Never smokers	Non-heavy smokers	Heavy smokers	*P*-value
	*N* = 1,192	*N* = 377	*N* = 113	*N* = 702	
ORR, *n* (%)					<0.001
Non-response	701 (58.8)	264 (70.0)	62 (54.9)	375 (53.4)	
Response	491 (41.2)	113 (30.0)	51 (45.1)	327 (46.6)	
DCR, *n* (%)					0.012
Non-response	207 (17.4)	83 (22.0)	20 (17.7)	104 (14.8)	
Response	985 (82.6)	294 (78.0)	93 (82.3)	598 (85.2)	
PFS					0.001
Median (95% CI), months	13.6 (12.2, 15.4)	11.2 (9.8, 12.8)	11.9 (9.9, 28.33)	15.9 (13.8, 17.4)	
% at 12 months (95% CI)	53.8 (50.6, 57.1)	46.1 (40.8, 52.1)	47.6 (37.9, 59.8)	58.9 (54.9, 63.3)	
% at 36 months (95% CI)	27.6 (24.2, 31.3)	22.6 (17.2, 28.8)	32.3 (22.5, 46.6)	29.9 (25.5, 35.1)	
% at 60 months (95% CI)	18.8 (14.4, 24.5)	11.4 (5.5, 23.6)	12.9 (4.2, 40.2)	24.1 (18.4, 31.4)	

ORR, objective response rate; DCR, disease control rate; PFS, progression-free survival; CI, confidence interval.

**Figure 3:**
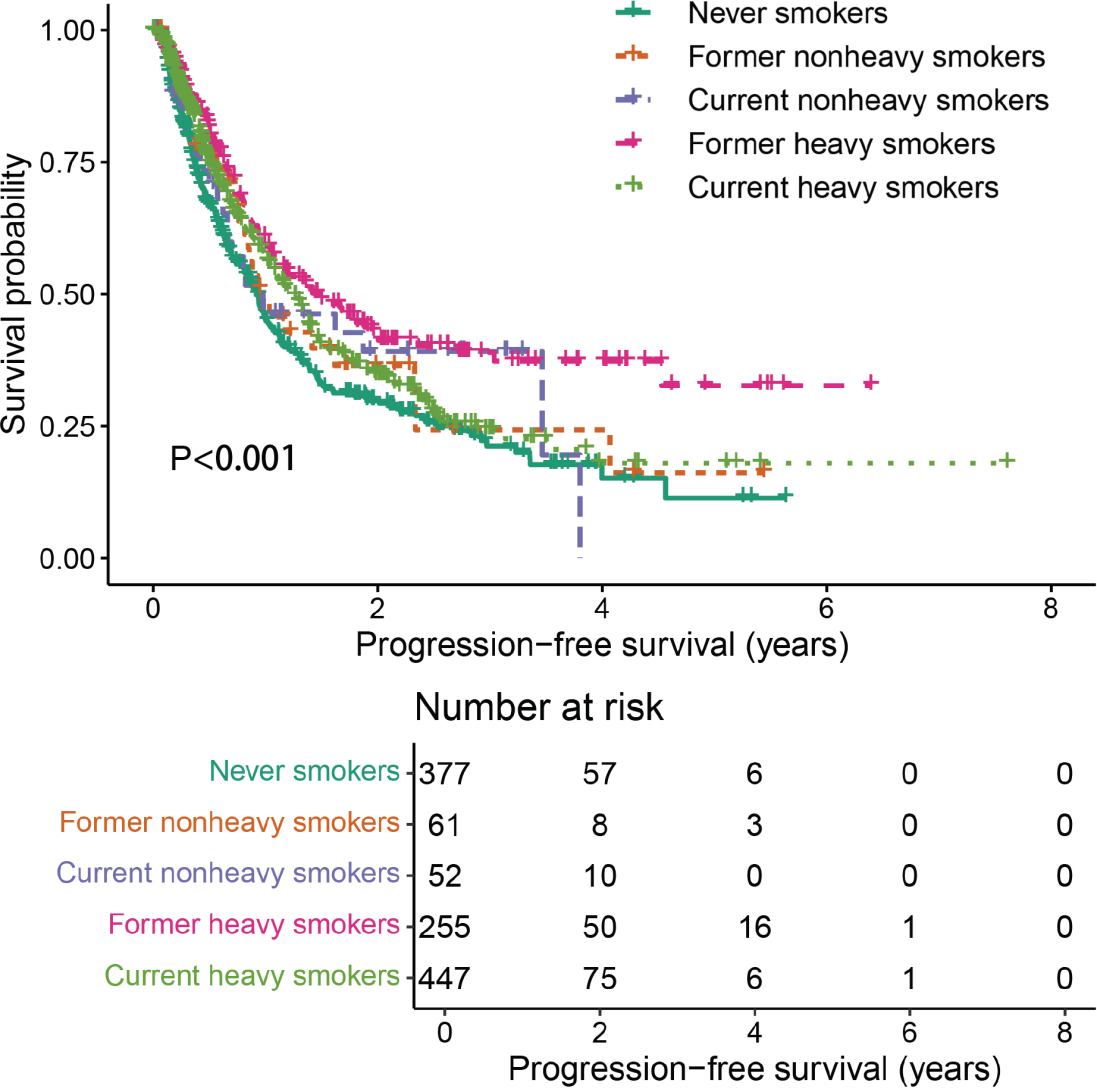
Comparison of stratified by smoking status and cumulative smoking dose.

Further analysis was performed to evaluate the combined effects of smoking status and cumulative smoking dose. Compared with never smokers, former heavy smokers demonstrated significantly enhanced PFS (*P* < 0.001, **Figure 3**).

### 3.4 Analysis of prognostic factors for ORR and PFS

For smoking status, multivariate analysis after adjusting for baseline covariates revealed that it was significantly associated with increased ORR. Current smokers exhibited an OR of 1.65 (95% CI: 1.12–2.45, *P* = 0.01), while former smokers had an OR of 2.01 (95% CI: 1.32–3.05, *P* = 0.001) (**Supplementary Table 1**). However, compared with never smokers, no significant differences in PFS were observed in current smokers (HR = 1.11, 95% CI: 0.87–1.42, *P* = 0.38) and former smokers (HR = 0.82, 95% CI: 0.63–1.06, *P* = 0.13) (**Supplementary Table 1**).

With respect to cumulative smoking dose, both non-heavy smokers (OR = 1.70, 95% CI: 1.01–2.85, *P* = 0.04) and heavy smokers (OR = 1.80, 95% CI: 1.23–2.64, *P* = 0.002) demonstrated significantly improved ORR compared with never smokers. However, cumulative smoking dose was not independently associated with PFS in patients with advanced NSCLC receiving immunotherapy (non-heavy smokers: HR = 1.14, 95% CI: 0.82–1.58, *P* = 0.44; heavy smokers: HR = 0.95, 95% CI: 0.75–1.20, *P* = 0.67) (**Supplementary Table 2**).

Further analysis, incorporating both smoking status and cumulative smoking dose, revealed that former heavy smokers were significantly associated with improved ORR (OR = 1.93, 95% CI: 1.25–2.99, *P* = 0.003) and prolonged PFS (HR = 0.75, 95% CI: 0.57–0.99, *P* = 0.04). These findings suggest that former heavy smoking is an independent protective factor for better ORR and PFS in patients receiving immunotherapy (**Table 4**).

**Table 4:** Association between smoking status, cumulative smoking dose, ORR, and PFS.

Characteristic	ORR				PFS			
	Univariable analysis		Multivariable analysis		Univariable analysis		Multivariable analysis	
	OR (95% CI)	*P*	OR (95% CI)	*P*	HR (95% CI)	*P*	HR (95% CI)	*P*
Gender (female ref.)								
Male	1.83 (1.33, 2.51)	<0.001	0.84 (0.53, 1.33)	0.45	0.66 (0.55, 0.79)	<0.001	0.82 (0.62, 1.07)	0.14
Age (<60 ref.)								
≥60	1.14 (0.91, 1.44)	0.26			0.69 (0.59, 0.81)	<0.001	0.73 (0.62, 0.86)	<0.001
BMI (<18.5 ref.)								
18.5–24.9	1.42 (0.93, 2.17)	0.11			0.89 (0.67, 1.16)	0.38		
≥25	1.45 (0.91, 2.31)	0.12			0.91 (0.67, 1.23)	0.55		
ECOG PS (0–1 ref.)								
≥2	0.60 (0.38, 0.96)	0.03	0.70 (0.42, 1.15)	0.15	1.84 (1.41, 2.40)	<0.001	1.79 (1.37, 2.34)	<0.001
Treatment line (1st ref.)								
More than 2nd	0.25 (0.19, 0.33)	<0.001	0.29 (0.22, 0.38)	<0.001	1.61 (1.38, 1.88)	<0.001	1.45 (1.23, 1.71)	<0.001
Histology (LUAD ref.)								
LUSC	1.57 (1.23, 2.00)	<0.001	1.04 (0.79, 1.38)	0.79	0.81 (0.68, 0.95)	0.01	1.00 (0.84, 1.20)	0.98
Others	1.30 (0.84, 1.99)	0.23	1.29 (0.81, 2.04)	0.29	0.95 (0.72, 1.25)	0.71	0.91 (0.69, 1.21)	0.51
Stage (III ref.)								
IV	0.42 (0.32, 0.53)	<0.001	0.54 (0.41, 0.71)	<0.001	1.96 (1.62, 2.39)	<0.001	1.74 (1.42, 2.14)	<0.001
Smoke (never smoker ref.)								
Former non-heavy smoker	2.75 (1.59, 4.77)	<0.001	2.31 (1.23, 4.33)	0.01	0.83 (0.57, 1.21)	0.33	1.12 (0.75, 1.68)	0.57
Current non-heavy smokers	1.24 (0.67, 2.28)	0.50	1.15 (0.57, 2.31)	0.69	0.81 (0.54, 1.22)	0.31	1.18 (0.76, 1.83)	0.45
Former heavy smokers	1.92 (1.38, 2.67)	<0.001	1.93 (1.25, 2.99)	0.003	0.61 (0.49, 0.77)	<0.001	0.75 (0.57, 0.99)	0.04
Current heavy smokers	2.11 (1.58, 2.81)	<0.001	1.72 (1.15, 2.56)	0.008	0.80 (0.67, 0.97)	0.02	1.10 (0.86, 1.41)	0.44
PD-L1 (<1% ref.)								
1%–49%	1.29 (0.87, 1.90)	0.20	1.16 (0.76, 1.77)	0.49	0.68 (0.54, 0.86)	0.001	0.70 (0.55, 0.89)	0.003
≥50%	2.08 (1.40, 3.10)	<0.001	1.74 (1.13, 2.67)	0.01	0.42 (0.31, 0.54)	<0.001	0.46 (0.35, 0.60)	<0.001
Not assessed	1.47 (1.03, 2.11)	0.03	1.17 (0.79, 1.72)	0.43	0.60 (0.48, 0.75)	<0.001	0.61 (0.49, 0.76)	<0.001

ORR, objective response rate; PFS, progression-free survival; OR, odds ratio; HR, hazard ratio; CI, confidence interval; BMI, body mass index; ECOG PS, Eastern Cooperative Oncology Group Performance Status; LUAD, lung adenocarcinoma; LUSC, lung squamous cell carcinoma; PD-L1, programmed cell death-ligand 1.

## 4 Discussion

This investigation represents the first real-world analysis to incorporate both smoking status and cumulative smoking dose in the evaluation of immunotherapy efficacy in patients with advanced NSCLC. Our findings suggest that immunotherapy efficacy is significantly greater in ever smokers compared to never smokers, with former heavy smokers emerging as an independent favorable predictor for both improved ORR and PFS in advanced NSCLC patients receiving immunotherapy.

Our study aligns with existing literature that underscores the relevance of smoking status in influencing immunotherapy outcomes (23, 24). This could be explained by the following reasons. Firstly, a significant dose–response relationship between smoking history and TMB has been established in advanced lung adenocarcinoma (25). Increased TMB correlates with enhanced CD8-positive T-cell infiltration, higher neoantigen load, and distinct immune response signatures, all of which contribute to improved outcomes with immunotherapy (26). Secondly, tobacco smoke contains a range of carcinogens, such as polycyclic aromatic hydrocarbons (PAHs) like benzo[a]pyrene (BaP) and aromatic amines, including 4-aminobiphenyl. Exposure to BaP has been shown to upregulate the mRNA and surface expression of PD-L1, which is mediated by aryl hydrocarbon receptor (AhR) (27, 28). A recent pooled analysis of 11 clinical trials has indicated that PD-L1 expression has an appreciable impact on clinical outcomes for patients with NSCLC treated with immunotherapy in the first- or second-line treatment setting (29). Furthermore, compared with smoking-related lung cancers, lung cancer in individuals who have never smoked (LCINS) presents a distinct genomic landscape (19, 30). In the Asian NSCLC population, EGFR mutations occur in 40%–60% of cases, especially among never smokers (31). However, EGFR-mutant tumors are often represented as exhibiting an immune-cold phenotype, characterized by an immunosuppressive microenvironment and reduced immunogenicity, leading to poor response to PD-1/PD-L1 monotherapy (32, 33).

Notably, our data demonstrated that former heavy smokers represent a distinct subgroup with particularly favorable outcomes, highlighting the importance of smoking cessation, even following a history of heavy smoking. Our findings complement the existing literature by adding to the understanding of the impact of smoking cessation on improving the efficacy of immunotherapy. These findings are similar to those of the KEYNOTE-024 trial (6, 34), which demonstrated that pembrolizumab significantly prolonged OS compared to chemotherapy in former smokers (HR = 0.59, 95% CI: 0.41–0.85). In contrast, the efficacy of immunotherapy was superior but insignificant in current smokers (HR = 0.81, 95% CI: 0.41–1.60) and never smokers (HR = 0.90, 95% CI: 0.11–7.59) (34). A possible explanation for the observed differences may involve multiple mechanisms. First, smoking-associated oxidative stress activates the inflammatory response pathway, leading to further generation of reactive oxygen species (ROS) (35). Oxidative metabolism and hypoxia influence the immune TME through multiple mechanisms, thereby weakening the beneficial effects of immune checkpoint blockade therapy (36). Second, nicotine in current smokers may enhance tumor proliferation and angiogenesis and compromise the therapeutic effects of treatment via the nicotinic acetylcholine receptor (nAChR) signaling pathway (37). Additionally, exposure to tobacco smoke constituents may upregulate the expression of immunosuppressive proteins, such as YAP1 (38, 39). These findings may indicate the importance of early smoking cessation to optimize immunotherapy strategies.

Nevertheless, our study has several inherent limitations. First, as a retrospective analysis, it is susceptible to selection bias. Additionally, because it depends on the accuracy and completeness of existing data, there may be issues with missing or inaccurate information. Second, OS data are not yet available due to the relatively early stage of follow-up at the time of analysis. We plan to continue monitoring OS in subsequent follow-up assessments, with results to be reported once the data reach sufficient maturity. Third, given that the data collection period spanned the COVID-19 pandemic, the lack of systematic recording of patients’ COVID-19 infection status limited our ability to evaluate its potential impact on treatment efficacy. Finally, while the strengths of the current study include a large sample size with high follow-up rates, further larger-scale, multicenter prospective studies are needed to validate our findings and explore the underlying molecular mechanisms, including the impact of different genetically driven mutations.

## 5 Conclusions

Our study demonstrates former heavy smokers (≥20 pack-years) as an independent favorable prognostic factor for advanced NSCLC patients receiving immunotherapy. These findings underscore the importance of early cessation in clinical settings. In addition, clinical assessments should prioritize the standardized documentation of detailed smoking history, including smoking status and cumulative smoking dose. Further prospective studies are needed to validate its clinical utility and explore the underlying biological mechanisms, with the aim of optimizing immunotherapy outcomes in patients with advanced NSCLC.

## Data Availability

The raw data supporting the conclusions of this article will be made available by the authors, without undue reservation.
