# Supplementary material for: Prognostic stratification through smoking status and cumulative smoking dose in advanced non-small cell lung cancer immunotherapy: a dose-dependent real-world analysis
Source: Front Oncol. 2025 Aug 6;15:1590825. doi: 10.3389/fonc.2025.1590825 (PMC12364628; doi:10.3389/fonc.2025.1590825)
Supplement: Supplementary file 1 [file Table1.docx]

Supplementary Material

# Supplementary Table 1. Association between smoking status and ORR, PFS.

| **Characteristic** | **ORR** | | |  | **PFS** | | | |
| --- | --- | --- | --- | --- | --- | --- | --- | --- |
|  | Univariable analysis | | Multivariable analysis | | Univariable analysis |  | Multivariable analysis |  |
|  | OR (95%CI) | P | OR (95%CI) | P | HR (95%CI) | P | HR (95%CI) | P |
| Gender (Female ref.) |  |  |  |  |  |  |  |  |
| Male | 1.83 (1.33, 2.51) | <0.001 | 0.83 (0.52, 1.33) | 0.44 | 0.66 (0.55, 0.79) | <0.001 | 0.81 (0.62, 1.06) | 0.12 |
| Age (<60 ref.) |  |  |  |  |  |  |  |  |
| ≥60 | 1.14 (0.91, 1.44) | 0.26 |  |  | 0.69 (0.59, 0.81) | <0.001 | 0.73 (0.62, 0.86) | <0.001 |
| BMI (<18.5 ref.) |  |  |  |  |  |  |  |  |
| 18.5-24.9 | 1.42 (0.93, 2.17) | 0.11 |  |  | 0.89 (0.67, 1.16) | 0.38 |  |  |
| ≥25 | 1.45 (0.91, 2.31) | 0.12 |  |  | 0.91 (0.67, 1.23) | 0.55 |  |  |
| ECOG PS (0-1 ref.) |  |  |  |  |  |  |  |  |
| ≥2 | 0.60 (0.38, 0.96) | 0.03 | 0.70 (0.42, 1.15) | 0.16 | 1.84 (1.41, 2.40) | <0.001 | 1.77 (1.35, 2.31) | <0.001 |
| Treatment line (1st ref.) |  |  |  |  |  |  |  |  |
| more than 2nd | 0.25 (0.19, 0.33) | <0.001 | 0.28 (0.22, 0.37) | <0.001 | 1.61 (1.38, 1.88) | <0.001 | 1.43 (1.22, 1.69) | <0.001 |
| Histology (LUAD ref.) |  |  |  |  |  |  |  |  |
| LUSC | 1.57 (1.23, 2.00) | <0.001 | 1.04 (0.78, 1.37) | 0.81 | 0.81 (0.68, 0.95) | 0.01 | 1.00 (0.83, 1.20) | 0.99 |
| Others | 1.30 (0.84, 1.99) | 0.23 | 1.29 (0.81, 2.04) | 0.28 | 0.95 (0.72, 1.25) | 0.71 | 0.93 (0.70, 1.23) | 0.62 |
| Stage (III ref.) |  |  |  |  |  |  |  |  |
| IV | 0.42 (0.32, 0.53) | <0.001 | 0.54 (0.41, 0.71) | <0.001 | 1.96 (1.62, 2.39) | <0.001 | 1.74 (1.42, 2.13) | <0.001 |
| Smoke (Never smoker ref.) |  |  |  |  |  |  |  |  |
| Current smoker | 2.00 (1.51, 2.65) | <0.001 | 1.65 (1.12, 2.45) | 0.01 | 0.81 (0.67, 0.96) | 0.02 | 1.11 (0.87, 1.42) | 0.38 |
| Former smoker | 2.06 (1.51, 2.81) | <0.001 | 2.01 (1.32, 3.05) | 0.001 | 0.65 (0.53, 0.80) | <0.001 | 0.82 (0.63, 1.06) | 0.13 |
| PD-L1 (<1% ref.) |  |  |  |  |  |  |  |  |
| 1-49% | 1.29 (0.87, 1.90) | 0.20 | 1.17 (0.77, 1.77) | 0.47 | 0.68 (0.54, 0.86) | 0.001 | 0.69 (0.55, 0.88) | 0.002 |
| ≥50% | 2.08 (1.40, 3.10) | <0.001 | 1.74 (1.13, 2.68) | 0.01 | 0.42 (0.31, 0.54) | <0.001 | 0.46 (0.35, 0.59) | <0.001 |
| Not assessed | 1.47 (1.03, 2.11) | 0.03 | 1.17 (0.79, 1.72) | 0.43 | 0.60 (0.48, 0.75) | <0.001 | 0.61 (0.49, 0.76) | <0.001 |

# Supplementary Table 2. Association between cumulative smoking dose and ORR, PFS.

| **Characteristic** | **ORR** | | |  | **PFS** | | | |
| --- | --- | --- | --- | --- | --- | --- | --- | --- |
|  | Univariable analysis | | Multivariable analysis | | Univariable analysis |  | Multivariable analysis |  |
|  | OR (95%CI) | P | OR (95%CI) | P | HR (95%CI) | P | HR (95%CI) | P |
| Gender (Female ref.) |  |  |  |  |  |  |  |  |
| Male | 1.83 (1.33, 2.51) | <0.001 | 0.83 (0.52, 1.32) | 0.43 | 0.66 (0.55, 0.79) | <0.001 | 0.81 (0.62, 1.06) | 0.13 |
| Age (<60 ref.) |  |  |  |  |  |  |  |  |
| ≥60 | 1.14 (0.91, 1.44) | 0.26 |  |  | 0.69 (0.59, 0.81) | <0.001 | 0.73 (0.62, 0.86) | <0.001 |
| BMI (<18.5 ref.) |  |  |  |  |  |  |  |  |
| 18.5-24.9 | 1.42 (0.93, 2.17) | 0.11 |  |  | 0.89 (0.67, 1.16) | 0.38 |  |  |
| ≥25 | 1.45 (0.91, 2.31) | 0.12 |  |  | 0.91 (0.67, 1.23) | 0.55 |  |  |
| ECOG PS (0-1 ref.) |  |  |  |  |  |  |  |  |
| ≥2 | 0.60 (0.38, 0.96) | 0.03 | 0.70 (0.43, 1.16) | 0.17 | 1.84 (1.41, 2.40) | <0.001 | 1.77 (1.35, 2.31) | <0.001 |
| Treatment line (1st ref.) |  |  |  |  |  |  |  |  |
| more than 2nd | 0.25 (0.19, 0.33) | <0.001 | 0.29 (0.22, 0.38) | <0.001 | 1.61 (1.38, 1.88) | <0.001 | 1.38 (1.18, 1.63) | <0.001 |
| Histology (LUAD ref.) |  |  |  |  |  |  |  |  |
| LUSC | 1.57 (1.23, 2.00) | <0.001 | 1.04 (0.79, 1.38) | 0.77 | 0.81 (0.68, 0.95) | 0.01 | 1.00 (0.84, 1.20) | 0.99 |
| Others | 1.30 (0.84, 1.99) | 0.23 | 1.29 (0.81, 2.05) | 0.28 | 0.95 (0.72, 1.25) | 0.71 | 0.93 (0.70, 1.24) | 0.63 |
| Stage (III ref.) |  |  |  |  |  |  |  |  |
| IV | 0.42 (0.32, 0.53) | <0.001 | 0.54 (0.41, 0.71) | <0.001 | 1.96 (1.62, 2.39) | <0.001 | 1.73 (1.41, 2.12) | <0.001 |
| Smoke (Never smoker ref.) |  |  |  |  |  |  |  |  |
| Nonheavy smokers | 1.92 (1.25, 2.96) | 0.003 | 1.70 (1.01, 2.85) | 0.04 | 0.82 (0.61,1.10) | 0.18 | 1.14 (0.82, 1.58) | 0.44 |
| Heavy smokers | 2.04 (1.56, 2.66) | <0.001 | 1.80 (1.23, 2.64) | 0.002 | 0.73 (0.62, 0.86) | <0.001 | 0.95 (0.75, 1.20) | 0.67 |
| PD-L1 (<1% ref.) |  |  |  |  |  |  |  |  |
| 1-49% | 1.29 (0.87, 1.90) | 0.20 | 1.17 (0.77, 1.77) | 0.47 | 0.68 (0.54, 0.86) | 0.001 | 0.69 (0.54, 0.87) | 0.002 |
| ≥50% | 2.08 (1.40, 3.10) | <0.001 | 1.74 (1.13, 2.67) | 0.01 | 0.42 (0.31, 0.54) | <0.001 | 0.46 (0.35, 0.59) | <0.001 |
| Not assessed | 1.47 (1.03, 2.11) | 0.03 | 1.17 (0.79, 1.72) | 0.43 | 0.60 (0.48, 0.75) | <0.001 | 0.60 (0.48, 0.75) | <0.001 |

Abbreviation: ORR, Objective response rate; PFS, Progression-free survival; OR, odds ratio; HR, hazard ratio; CI, confidence interval; BMI, body mass index; ECOG PS, Eastern Cooperative Oncology Group Performance Status; LUAD, Lung adenocarcinoma; LUSC, Lung squamous cell carcinoma; PL-L1, programmed cell death-ligand 1.
